# Newly Diagnosed Patients with Inflammatory Bowel Disease: The Relationship Between Perceived Psychological Support, Health-Related Quality of Life, and Disease Activity

**DOI:** 10.1089/heq.2020.0053

**Published:** 2021-02-02

**Authors:** Kristy Engel, Maher Homsi, Rie Suzuki, Karla Helvie, Jeremy Adler, Caitlyn Plonka, Ellen Zimmermann

**Affiliations:** ^1^ Department of Public Health and Health Sciences, College of Health Sciences, University of Michigan-Flint, Flint, Michigan, USA.; ^2^ Division of Gastroenterology, Department of Internal Medicine, University of Florida College of Medicine, Gainesville, Florida, USA.; ^3^ Department of Internal Medicine, Division of Gastroenterology, University of Michigan Medical School, University of Michigan, Ann Arbor, Michigan, USA.; ^4^ Division of Pediatrics Gastroenterology, Department of Pediatrics and Communicable Diseases, University of Michigan Medical School, Ann Arbor, Michigan, USA.

**Keywords:** educational support, health-related quality of life, inflammatory bowel disease, psychological support

## Abstract

**Background:** Newly diagnosed patients with inflammatory bowel disease (IBD) encounter many physical, mental, and social uncertainties. In other chronic diseases, patients having access to disease-specific information and psychological support adhere better to medical regimens. Currently, there is a paucity of data on how newly diagnosed patients with IBD interact with their medical providers.

**Methods:** Patients diagnosed with IBD within 5 years completed a series of questionnaires related to heath-related quality of life (HRQoL), disease activity, health education resources, medical provider relationship, and psychological support.

**Results:** A total of 89 patients were included in the study. IBD activity correlated with disease-specific quality of life (*r*=−0.69, *p*<0.0001). Patient satisfaction with gastroenterologist interaction correlated with HRQoL (*r*=0.33, *p*=0.04) and disease activity for Crohn's disease (CD) patients (Harvey Bradshaw Index, *r*=−0.52, *p*<0.001). Eleven percent of recently diagnosed patients reported receiving educational or psychological support as part of their treatment program, whereas 42% of patients believed that they would benefit from having these types of support incorporated in their treatment protocol.

**Discussion:** In patients with newly diagnosed CD, the patients' perceived relationship with their medical provider was closely related to both HRQoL and disease activity. More attention to education, support, and the doctor–patient relationship at diagnosis could result in better patient outcomes.

## Introduction

Patients with newly diagnosed inflammatory bowel disease (IBD; Crohn's disease [CD] and ulcerative colitis [UC]) have questions and are looking for answers. Similar to most patients with other chronic illnesses, they access a variety of health-related sources; however, the most relevant personalized health information should come from their primary care physician, their gastroenterologist, and other medical professionals with whom they interact. It is not uncommon for patients with IBD to experience significant psychological and social stress associated with their chronic disease diagnosis thereby contributing to a reduction in their health-related quality of life (HRQoL).^1–3^ Interestingly, lowered HRQoL and persistent psychological distress can exist despite experiencing clinical remission; therefore, it is likely other variables separate from disease activity influence a patient's HRQoL.^4–8^ We investigated the possibility that the doctor–patient relationship and access to educational and psychological support affect patients' HRQoL.

Patients adjusting to their IBD diagnosis will benefit from resources provided by medical professionals. The early months after diagnosis are particularly challenging for patients with IBD because of the complexity of the medical information, testing, medication adjustments, and therapeutic strategies being discussed and implemented. It has been shown that patients with greater knowledge about their disease tend to have more adaptive coping strategies.^9^ Furthermore, patients with a better knowledge base tend to have better adherence to medical regimens leading to better patient outcomes.^10^

Psychological support is an important dimension of patient care, particularly early in the disease course. In oncology, cardiology, and rheumatology care, social support has become a standard part of patient treatment protocols as this support improves performance status and adherence to medication plans.^11^ Resources received by patients with IBD from a medical professional during an educational or psychological support session have the potential to positively impact how a patient copes with the diagnosis.^12^

To our knowledge, no studies have focused on patient's satisfaction with available psychological and educational support, disease education, and the relationship with a gastroenterologist early in the disease course. We hypothesized that patients who are satisfied with their educational and psychological support will report a higher HRQoL and demonstrate improved disease activity.

## Materials and Methods

Ninety-four patients were recruited from the University of Michigan Health System Inflammatory Bowel Disease Clinic. Patients were eligible if they were ≥14 years, if they fulfilled the diagnostic criteria for IBD provided and verified by their treating gastroenterologist, and if they had been diagnosed with IBD within 5 years of the study's commencement. The diagnosis was confirmed by standard diagnostic criteria. Out of the 94 patients who were recruited, one patient withdrew after starting the survey. Two additional surveys were eliminated due to the patients' diagnosis date not being confirmed. Two surveys were only partially completed. Therefore, 89 completed surveys were analyzed.

Excluded from the study were patients with multiple health complications not related to IBD, and patients who had difficulty understanding and/or speaking English. Informed consents were obtained from all patients. Patients not being seen for an office visit during the time of the face-to-face questionnaire distribution were contacted by mail. Participants received an incentive of five dollars for participating in the study. This study was approved by the University of Michigan Institutional Review Board (HUM00047447).

Demographic questions were asked that pertained to age, gender, race, ethnicity, marital status, educational level, and employment status. Questions aimed to categorize patients into IBD categories of UC, CD, or indeterminate colitis in addition to obtaining information on the month and year of IBD diagnosis.

### Survey instruments

The questionnaire utilized in the study comprised original and modified questions from the Short-Form Inflammatory Bowel Disease Questionnaire (SIBDQ)^13^ and the Medical Outcomes Study Social Support Survey (MOS).^14^ The SIBDQ is a tool that measures HRQoL in patients with IBD. The MOS was modified to include questions pertaining to the patients' interaction with their gastroenterologists, and is, therefore, noted in the study results as The Modified Medical Outcomes Study Social Support Survey (GI-MOS). Lower GI-MOS scores represent a decrease in satisfaction with gastroenterologist. Disease activity was measured utilizing the Harvey Bradshaw Index (HBI),^15^ and the Simple Clinical Colitis Activity Index (SCCAI).^16^ Higher scores indicate more disease activity. Finally, patient demographic questions were adapted from the Center for Disease Control and Prevention's Behavioral Risk Surveillance System.

### Statistical analysis

Descriptive statistics were used to characterize the population's demographics. The Spearman rank correlation was calculated to investigate the impacts of disease activity (HBI and SCCAI) on HRQoL (SIBDQ) and the impacts of patients' satisfaction with gastroenterologist (GI-MOS) on disease activity (HBI and SCCAI) and HRQoL (SIBDQ). The Spearman rank correlation was used because both the GI-MOS and SIBDQ provide ordinal results and thus require nonparametric statistic. The data were sorted and calculated using Microsoft Excel and the Statistical Packages for the Social Sciences (SPSS) software program (version 24; IBM SPSS, Inc.). A *p*-value ≤0.05 was considered statistically significant.

## Results

Patient demographics and therapy are shown in Tables 1 and 2. A majority of patients had been diagnosed within the past 1–2 years (55%). Table 3 displays patients' surgery history. Twenty patients had surgery history (15 patients with CD and 5 patients with UC). Most common surgeries were intestinal resection (6) or abscess drainage (6).

**Table 1.** Demographic Data of Study Cohort

	*n* (%)
Demographic characteristics	89
Age (mean in years)	37.63 (SD=16.87)
Gender (male:female)	46:45 (51:49)
Ethnicity (1=white)	86 (96)
Relationship status	
Married	44 (49)
Divorced	6 (7)
Widowed	2 (2)
Separated	1 (1)
Never married	36 (40)
Disease diagnosis	
UC	53 (60)
CD	36 (40)
Disease duration	
0–3 months	3 (3)
4–6 months	9 (10)
7–9 months	10 (11)
10–12 months	7 (8)
1–2 years	49 (55)
3–5 years	11 (12)

CD, Crohn's disease; SD, standard deviation; UC, ulcerative colitis.

**Table 2.** Medications Taken by Patients Participating in the Survey

Group	No. of patients, *n* (%)
Biologics-based therapy	18 (19)
+no other medications	8
+immunomodulators	5
+5-ASA products+steroids	5
Immunomodulators-based therapy	34 (37)
+no other medications	12
+5-ASA products	8
+steroids	7
+5-ASA products+steroids	4
+other immunomodulators+steroids	1
+allopurinol	2
Steroid-based therapy	32 (34)
5-ASA+steroids	31
Corticosteroids alone	1
No medications	7 (8)
Other	2
Double-blind clinical trial (Remicade vs. placebo after surgery)	2

Biologics: anti-TNF therapy and vedolizumab; immunomodulators: azathioprine, mercaptopurine, and tacrolimus; 5-ASA product: any mesalamine containing product; steroids: prednisone, budesonide, or rectal steroids.

**Table 3.** Surgical History of Patients Participating in the Study

Surgery	CD	UC
Colectomy	2	2
Ileostomy	0	2
Ileoanal pouch anastomosis	0	1
Fistula surgery	1	0
Abscess drainage	6	0
Intestinal resection	6	0
Total	15	5

**FIG. 1.**
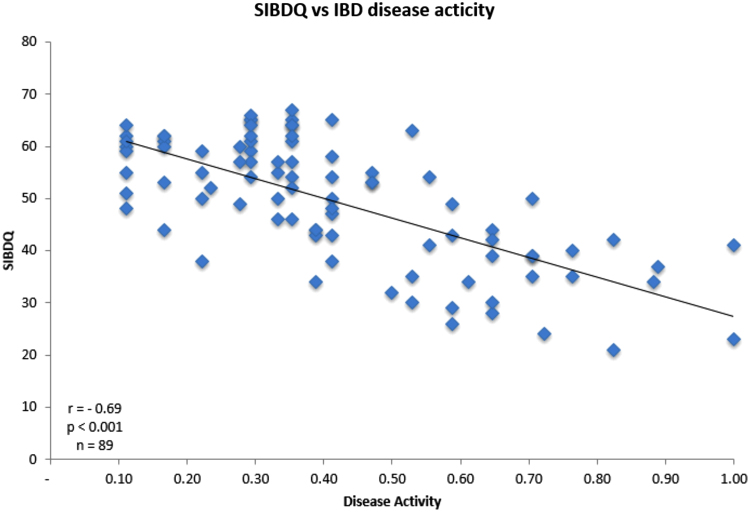
The relationship between and the SIBDQ and combined disease activity of UC and CD. CD, Crohn's disease; SIBDQ, short-form inflammatory bowel disease questionnaire; UC, ulcerative colitis.

As expected,^17^ the patients' HRQoL correlated with disease activity for both CD (HBI) and UC (SCCAI). IBD patients with active disease have a reduced HRQoL (SIBDQ, *r*=−0.69, *p*<0.0001, *n*=89; Fig. 1).

**FIG. 2.**
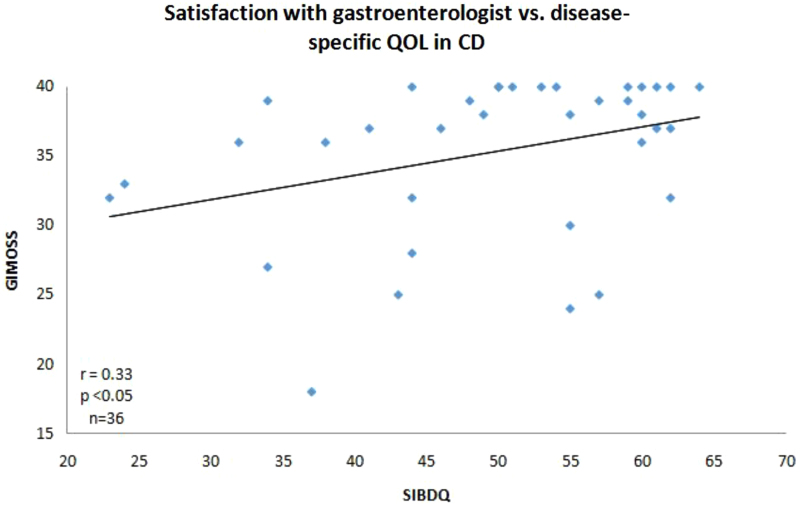
The relationship between patient's satisfaction with gastroenterologist and the SIBDQ in patients with CD. GI-MOS, The Modified Medical Outcomes Study Social Support Survey; QOL, quality of life.

**FIG. 3.**
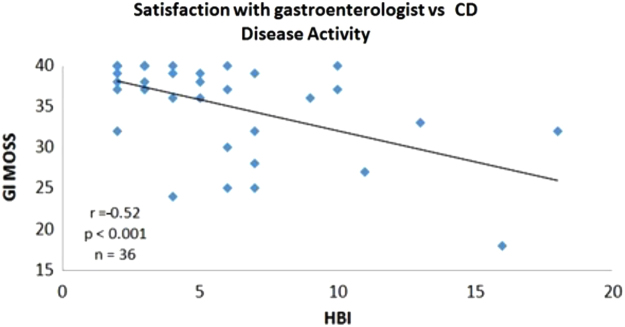
The relationship between patient's satisfaction with gastroenterologist and disease activity in patients with CD. HBI, Harvey Bradshaw Index.

We used a modified MOS (GI-MOS) to incorporate questions pertaining to a patient's perception of his or her gastroenterologist (Table 4). Patients with IBD who reported higher level of satisfaction with their gastroenterologist (GI-MOS) also reported a higher HRQoL. When analyzed separately, GI-MOS correlated positively with HRQoL for CD but not for UC (GI-MOS vs. SIBDQ for CD: *r*=0.33, *p*=0.04, *n*=36; GI-MOS vs. SIBDQ for UC: *r*=0.15, *p*=0.27, *n*=53; Fig. 2). CD patients with more active disease had lower satisfaction with their gastroenterologist (GI-MOS). GI-MOS correlated negatively with disease activity in CD but not UC (GI-MOS vs. HBI, *r*=−0.53, *p*<0.001, *n*=36; GI-MOS vs. SCCAI, *r*=−0.03, *p*=0.80, *n*=53; Fig. 3).

**Table 4.** The Modified Medical Outcomes Study Social Support Survey

1. Your gastroenterologist is someone you can count on to listen to you when you need to talk about your IBD diagnosis
2. Your gastroenterologist is someone to give you good advice about your IBD diagnosis
3. Your gastroenterologist is someone to give you information to help you understand your IBD diagnosis
4. Your gastroenterologist is someone to confide in or talk to about yourself or your IBD problems
5. Your gastroenterologist is someone whose advice you really want
6. Your gastroenterologist is someone to share your most private worries and fears about your IBD with
7. Your gastroenterologist is someone to turn to for suggestions about how to deal with your IBD
8. Your gastroenterologist is someone who understands your problems

All questions had the following answers to pick from (none, a little, some, most, or all of the time).

IBD, inflammatory bowel disease.

### Perceived psychological support

**FIG. 4.**
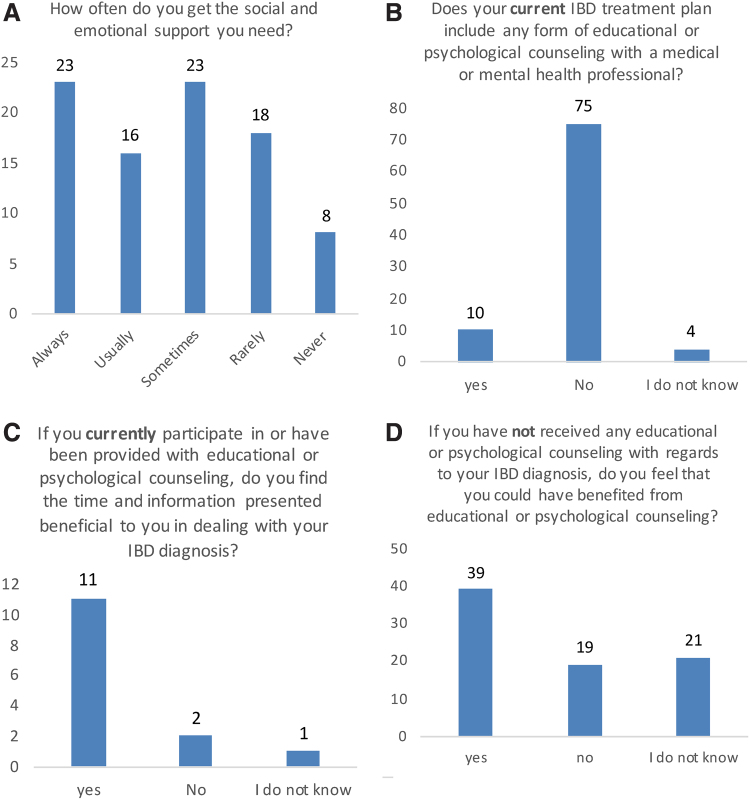
**(A)** How often do you get the social and emotional support you need? **(B)** Does your current IBD treatment plan include any form of educational or psychological counseling with a medical or mental health professional? **(C)** If you currently participate in or have been provided with educational or psychological counseling, do you find the time and information presented beneficial to you in dealing with your IBD diagnosis? **(D)** If you have not received any educational or psychological counseling with regard to your IBD diagnosis, do you feel that you could have benefited from educational or psychological counseling? IBD, inflammatory bowel disease.

Patients were questioned on psychological support in their lives in general and in their IBD treatment plans. Patients reporting rarely and never receiving psychological support had the most active CD (*r*=0.33, *p*=0.043, *n*=36). Forty-nine patients (56%) reported receiving these forms of support on either an intermittent basis to never (Fig. 4A). Only 11% of the patient population reported having educational and psychological support as part of their current treatment plan (Fig. 4B). Eleven patients (80%) of those receiving educational and psychological support in their treatment answered that it was beneficial (Fig. 4C). Forty-nine percent of patients (39) who have not received educational or psychological counseling regarding IBD diagnosis feel that they could have benefited from it (Fig. 4D).

## Discussion

A major finding of this study is a correlation between patients' relationship with their gastroenterologist and their overall HRQoL. This finding highlights the need for patients to feel comfortable in their relationship with the treating gastroenterologist. A good bidirectional relationship is critical for eliciting pertinent historical information, effectively communicating disease-related information, comfortably asking questions and expressing concerns, and cooperating in decision making regarding testing and treatment. Communicating potentially sensitive information regarding mental health, sexuality, or substance use/abuse also relies on a good working relationship. Creating a safe atmosphere where patients are encouraged to be open and forthright about supplemental therapies that the physician may or may not favor is also important.

In our study, a shockingly high percentage of recently diagnosed patients (80%) perceived little or no psychological support in their treatment plan. This suggests that IBD patients may have unmet mental health needs, which could hinder overall treatment. In CD, the patients who reported little or no psychological support had the most active CD. Although we cannot determine from our study whether this association is causative or whether patients who are more ill are less likely to acknowledge support or are biased in their assessments, the connection is important and warrants further study.

In our study, the noted associations with disease activity were observed for CD but not UC. CD symptoms are more likely to include abdominal pain compared with UC that favors diarrhea as a major symptom. It is likely that patients with pain have different exacerbating or alleviating factors that rely on a good relationship with their gastroenterologist and benefit from added psychological support. The associations were also stronger for the disease activity measure (HBI; Fig. 3) than the quality of life measure (SIBDQ; Fig. 2). This may reflect the characteristics of each measure, especially the nature and number of the measure's elements.

Our study is consistent with prior studies demonstrating the value of a multidisciplinary approach to IBD care.^18,19^ When surveyed, our study patients expressed the desire for educational and psychological support to be included in their treatment protocol although few acknowledged receiving this support. Furthermore, our study patients emphasized a desire to meet with a nurse, health educator, or counselor to discuss their diagnosis. Oliveira et al.^20^ and Ballou and Keefer^19^ emphasized the importance of addressing not only the physical demands of a chronic disease but also the psychosocial ramifications. Having the opportunity to discuss additional concerns regarding their IBD diagnosis with a health care professional would provide the social support for patients that could potentially influence their disease activity.^21^

The new avenues for health care information and psychological support on social media are constantly evolving. Data suggest that patients utilize social media for information, although disease-specific information is most commonly derived from their physicians. The sources of information and support are age dependent.^22^ Therefore, newly diagnosed IBD patients in their second or third decade of life are most likely to seek information and support on social media platforms that provide easily accessible interactions. The value of social medial relationships with respect to medical information and psychological support requires ongoing assessment.^22^

Our study has several limitations. Currently, there is no validated survey instrument focusing on patient satisfaction pertaining to gastroenterology care. Therefore, to measure this aspect, we modified the previously validated MOS survey. As with any survey modification, original validation becomes nullified; however, this modification proved to be a key piece of the research process and could potentially inform future research. The survey provided to patients was lengthy, including 61 survey questions plus additional questions associated with diagnosis (HBI, SCCAI). Long survey tools contribute to patient fatigue and hasty responses if interest and time begin to wane. In addition, the research was conducted in a tertiary care center that can influence generalizability.

Finally, the initial visits of a CD patient to the gastroenterologist's office are often lengthy and encompass discussions of gastrointestinal symptoms, test results, treatment options, potential disease- or medication-related complications, and health care maintenance issues. The first few visits after the diagnosis is made are particularly busy leaving little time to address psychological needs. This study highlights the patients' perception of the importance of psychological support. Our findings reinforce the importance of a team approach with interaction between the patient and several caregivers and encourages enlisting professional mental health services in some cases. Further research should be aimed at stratifying psychological needs based on risk assessment and patient's perception.

## Abbreviations Used

CD: Crohn's disease
HBI: Harvey Bradshaw Index
HRQoL: heath-related quality of life
IBD: inflammatory bowel disease
MOS: Medical Outcomes Study Social Support Survey
QOL: quality of life
SCCAI: Simple Clinical Colitis Activity Index
SD: standard deviation
SIBDQ: Short-Form Inflammatory Bowel Disease Questionnaire
SPSS: Statistical Packages for the Social Sciences
UC: ulcerative colitis
